# Supplementary material for: The Correlation between Periodontal Parameters and Cell-Free DNA in the Gingival Crevicular Fluid, Saliva, and Plasma in Chinese Patients: A Cross-Sectional Study
Source: J Clin Med. 2022 Nov 23;11(23):6902. doi: 10.3390/jcm11236902 (PMC9741438; doi:10.3390/jcm11236902)
Supplement: Supplementary file 1 [file jcm-11-06902-s001.zip › jcm-1993781-supplementary.pdf]

Table S1. Multivariate analysis of age, sex and clinical parameters on cfDNA concentrations

Parameter estimates (β value)[a]								
		Variables						
		Age	Sex	Clinical parameters				
Samples	Intercept	(years)	(M=1; F=0)	Mean PD	Max PD	BOP	Mean PI	(R^2 value)[b]
GCF	-399.4	6.417	-10.39	185.6				0.449
(ng/ul	(p < 0.001)	(p = 0.044)	(p = 0.815)	(p < 0.001)				
per 30-s	-172.3	2.870	-20.97		78.04			0.471
sample)	(p = 0.581)	(p = 0.387)	(p = 0.629)		(p < 0.001)			
	-189.7	8.500	-9.607			5.080		0.513
	(p = 0.031)	(p = 0.003)	(p = 0.817)			(p < 0.001)		
	-195.9	8.050	-25.29				206.2	0.392
	(p = 0.046)	(p = 0.015)	(p = 0.588)				(p < 0.001)	
Saliva	-94.28	3.177	-60.14	114.6				0.505
(ng/ml)	(p = 0.107)	(p = 0.080)	(p = 0.019)	(p < 0.001)				
	47.33	1.355	-66.12		7.053			0.497
	(p = 0.372)	(p = 0.487)	(p = 0.011)		(p < 0.001)			
	39.22	4.834	-59.29			2.794		0.501
	(p = 0.458)	(p = 0.006)	(p = 0.022)			(p < 0.001)		
	33.73	4.400	-68.58				119.9	0.420
	(p = 0.555)	(p = 0.024)	(p = 0.014)				(p < 0.001)	
Plasma	134.5	1.709	25.79	35.21				0.128
(ng/ml)	(p = 0.008)	(p = 0.265)	(p = 0.232)	(p = 0.019)				
	178.2	1.191	24.02		13.72			0.124
	(p < 0.001)	(p = 0.470)	(p = 0.268)		(p = 0.024)			
	180.5	2.672	26.51			0.4388		0.080
	(p < 0.001)	(p = 0.077)	(p = 0.232)			(p = 0.242)		
	177.7	2.425	24.41				25.02	0.084
	(p < 0.001)	(p = 0.118)	(p = 0.271)				(p = 0.189)	

Abbreviations: cfDNA, cell-free DNA; GCF, gingival crevicular fluid; PD, probing depth; BOP, bleeding on probing; PI, plaque index; M, male; F, female.

[a,b]Multiple linear regression; n = 81.
